# Complete Genome of *Salmonella enterica* Serovar Typhimurium Myophage Maynard

**DOI:** 10.1128/genomeA.00866-13

**Published:** 2013-12-19

**Authors:** Casey O. Tatsch, Thammajun L. Wood, Karthik R. Chamakura, Gabriel F. Kuty Everett

**Affiliations:** Center for Phage Technology, Texas A&M University, College Station, Texas, USAa; Department of Biochemistry and Molecular Biology, Pennsylvania State University, University Park, Pennsylvania, USAb

## Abstract

*Salmonella enterica* serovar Typhimurium is a pathogenic bacterium that has been a major concern for food and public safety. Phages infecting *S*. Typhimurium may prove to be useful therapeutics against this harmful bacterium. Here, we announce the complete genome of *S*. Typhimurium T4-like myophage Maynard and describe its features.

## GENOME ANNOUNCEMENT

*Salmonella enterica* serovar Typhimurium is a Gram-negative bacterium that is found in a wide variety of animals, typically in the intestinal tract. One of the main interests in *S*. Typhimurium is its pathogenicity when ingested by consuming or handling contaminated fish, beef, and poultry products (1, 2). The survival and impact of *S*. Typhimurium are largely attributed to the adaptive mechanisms of the organism to survive in the harsh environments in which food is packaged and shipped (3, 4). Additionally, the rise of antibiotic resistance is making it harder to treat *S*. Typhimurium infections (5). Consequently, the use of bacteriophages for the biocontrol and treatment of *Salmonella* serovars is gaining momentum.

Bacteriophage Maynard was isolated from a sewage sample collected in College Station, TX. Phage DNA was sequenced using 454 pyrosequencing at the Emory GRA Genome Center (Emory University, Atlanta, GA). Trimmed FLX Titanium reads were assembled to a single contig at 167.3-fold coverage using the Newbler assembler, version 2.5.3 (454 Life Sciences), at default settings. The contig was confirmed to be complete by PCR. Genes were predicted using GeneMarkS (6) and corrected using software tools available on the Center for Phage Technology (CPT) portal (https://cpt.tamu.edu/cpt-software/portal/). Electron microscopy was performed at the Microscopy and Imaging Center at Texas A&M University.

Maynard is a T4-like myophage with a 154.7-kb genome, a coding density of 92.8%, and a G+C content of 45.6%. The G+C content is high compared to the normal range of T4-like phages (35% to 43%) (7). Genome analysis and annotation of Maynard show 204 predicted coding sequences, of which 78 have a predicted function by BLASTp, InterProScan, and CDD searches (8–10).

The T4-like core genes encoding proteins related to replication, recombination, DNA packaging, morphogenesis, DNA biosynthesis, and lysis were identified. Genes for replication and recombination proteins include those encoding DNA polymerase, helicase, primase, ligase, sliding clamp holder and loader, recombination/repair endonucleases, Holliday junction resolvase, RecA, end-protector protein, and topoisomerase. Genes identified for DNA biosynthesis proteins were those encoding dCMP deaminase, dUTPase, thymidylate synthase, and ribonucleotide reductase subunits alpha and beta. DNA packaging proteins found were the small and large terminases and the portal protein. Structural proteins confirming the myophage morphology of Maynard and homing endonucleases typical of T4-like phages were also identified. Interestingly, unlike T4, the tailspike has a pectin lyase domain, presumably for biofilm depolymerization (11).

Unlike T4 and most T4-like phages, the large terminase of Maynard is interrupted by an intein (12, 13). InterProScan revealed a Hint (Hedgehog/intein) domain (InterPro accession no. IPR003587) and an intein splice site (InterPro accession no. IPR006141) disrupting the coding sequence. An intein is a self-splicing intervening polypeptide that that religates flanking exteins upon excision (14). The genome also contains a phosphate starvation-inducible protein, PhoH, typically found in marine phages and only 4% of nonmarine phages (15).

### Nucleotide sequence accession number.

The genome sequence of phage Maynard was contributed as accession no. KF669654 to GenBank.
